# Recent development and perspectives of virtual slides (VS) and telepathology in Europe

**DOI:** 10.1186/1746-1596-8-S1-S2

**Published:** 2013-09-30

**Authors:** Klaus Kayser, Stephan Borkenfeld, Gian Kayser

**Affiliations:** 1 UICC-TPCC, Institute of Pathology, Charite, Berlin, Germany; 2 International Academy of Telepathology (IAT), Heidelberg, Germany; 3 Institute of Pathology, University of Freiburg, Freiburg, Germany

## Background

The new telepathology and virtual slide (VS) technology undergo remarkable changes in development and implementation. What are the reasons? What do we have to expect in the near future?

## Present stage

Telepathology started in Europe in the 1980s. It was implemented in closed communication systems and focussed on frozen section service [1,2]. Open systems embedded in open source software replaced the early communication about 10 years ago. They became to age, and nearly all of them closed [3]. A new era started with the **m**edical **e**lectronic **c**ommunication **e**xpert **s**ystem (MECES, http://www.diagnomx.eu) and the **V**irtual **I**nternational **P**athology **I**nstitute (VIPI,http://www.diagnomx.eu/vipi) that combine an internal communication network with external information nodes. It uses acoustic and visual information transfer as well as information sources at different levels such as access to libraries, image content information analysis, and diagnosis assistants [3]. It also incorporates VS, which are available from different companies. Although delivered in non-congruent formats certain medical platforms (MECES) and open access scientific journals (journal of diagnostic pathology (http://www.diagnosticpathology.org)) can handle VS via their specific viewers. VS implementation in routine tissue – based diagnosis is on its way. Most companies try to specifically connect their VS scanners to laboratory information systems (LIS) and / or to digital radiology imaging systems (picture archiving and communication system (PACS)) [4,5]. VS are provided with their own specific image analyzing system that focuses on evaluation of suitable immunohistochemistry scores such as Her2_neu or hormone receptors in breast cancer. Obligatory VS standards are still missing [6,7].

## Expectations

The establishment of a mandatory VS standard related to PACS is on its way [8]. Recent development of so – called social forums (facebook, linkedin, youtube, etc.) has lead to new communication standards that permit the extension of open access and open software forums to external nodes. They can be considered as a communication system with internal structures {discussion groups, communication pathways (images, sounds, movies, functions), language, data sets, etc.} equipped with flexible communicative surface {interpretation, measurements, quality assurance, standardization, language translation, and others}. These information transfers can be switched on and off [9].

There are two different ways to incorporate VS in routine tissue – based diagnosis, namely (a) direct implementation of VS scanners in the existing LIS with specific connection to the hospital information system (HIS), and (b) to create an open communication network that provides as flexible communication surface to HIS, LIS, VS, etc. Industry seems to prefer method (a) although method (b) offers great advantages [10].

## Conclusion

Europe is involved in big changes that involve the world of tissue – based diagnosis [11]. Surgical pathology starts to gain in clinical significance and financial interest. It is promoted by predictive diagnosis and communicative approaches which have their roots in telemedicine and digitized images [12]. The digitalization of surgical pathology has irreversibly started with big investment; the out come of the footrace is promising; however, it remains still open.
